# Patterns of insomnia and its treatment in North Central London: using primary care data to establish unmet needs and health inequalities

**DOI:** 10.1192/bjo.2025.10861

**Published:** 2025-12-17

**Authors:** Lauren Z. Waterman, Fleur O. M. Harrison, Uche Osuagwu, Sarah Dougan

**Affiliations:** North Central London Integrated Care System, London, UK; Institute of Psychiatry, Psychology and Neuroscience, King’s College London, UK; North London NHS Foundation Trust, London, UK; Public Health Team, Islington Council, London, UK

**Keywords:** Sleep–wake disorders, insomnia, prevention, population health, sleep

## Abstract

**Background:**

Existing research demonstrates that insomnia is common, with significant negative impacts on health and quality of life. Cognitive–behavioural therapy for insomnia (CBT-I), the first-line treatment, is highly cost-effective. However, healthcare records have not been used in the UK to establish real-world insomnia prevalence, inequalities or unmet need.

**Aims:**

This study’s aim was to establish the above in North Central London.

**Method:**

Data were extracted from primary care records across three London boroughs for 765 035 patients. Prevalence was determined by identifying those with a recent code for insomnia, insomnia treatment or sleeping tablet prescription.

**Results:**

Insomnia prevalence was 4.3%. Prevalence increased steadily with age, and was highest for women (4.9%), those of Bangladeshi ethnicity (7.3%) and those in the most deprived quintile (5.2%). Prevalence was significantly higher in patients with comorbidities (including chronic obstructive pulmonary disease (17.5%), severe mental illness (16.6%) and depression (14.1%)). Only 1.7% of people with insomnia had been referred for CBT-I.

**Conclusions:**

Findings suggested that insomnia is at least as common as illnesses receiving high levels of focus and resourcing in the UK, and that prevalence estimates were probably underestimates. Variation in prevalence by demographic factors and deprivation may represent health inequalities. Insomnia was particularly common among patients with certain comorbidities and of advancing age, indicating that those groups should be actively screened. Concerningly, referral rates for CBT-I were extremely low. This has important implications regarding population health management, commissioning and training. Prevalence and unmet need are likely to be high in many other areas and should be investigated locally.

There is strong evidence from both national and international research that insomnia is highly prevalent in the general population, with a significant adverse impact on physical health, mental health and quality of life,^1^ and a high economic impact.^2^ For example, insomnia has repeatedly been shown to increase the risk of developing new mental illnesses (such as depression) in people with no psychiatric history, and to reduce the efficacy of treatment of comorbid mental disorders, exacerbate their severity and precipitate relapses.^3^ Whereas insomnia used to be mainly considered a consequence of disorders such as depression or chronic pain, it is no longer separated into ‘primary’ and ‘secondary’ insomnia due to the often bidirectional relationship between insomnia and other disorders, and the fact that research has consistently demonstrated the effectiveness of treating insomnia directly regardless of comorbidity or order in which the insomnia and comorbidity occurred.^1^ A significant body of high-quality research consistently demonstrates that cognitive–behavioural therapy for insomnia (CBT-I) leads to durable improvements in insomnia symptoms (around 60–80% experience clinically significant improvements),^4^ as well as in a wide range of comorbid conditions.^1^ This is particularly important for insomnia that has become chronic, since research suggests that chronic insomnia, which is classified as a mental disorder in many diagnostic manuals, rarely remits spontaneously.^5^ It is also highly cost-effective^2^ with a low burden on resources,^1^ leading to it becoming the first-line recommended treatment by bodies such as the American College of Physicians^6^ and the UK’s National Institute for Health and Clinical Excellence (NICE), which recommends it for insomnia that has become chronic or is likely to become chronic.^7^ However, in many countries, including the UK,^1^ USA^8,9^ and Western European countries,^10^ chronic insomnia is known to be under-treated due to a paucity of available services providing CBT-I and lack of awareness of the treatment. In the UK, psychoeducation on ‘sleep hygiene’ is more commonly provided; however, research shows that this is ineffective for most people.^1,11^

Previous research into insomnia prevalence has mostly revolved around cross-sectional and longitudinal survey studies,^9,12^ and suggests that its prevalence in England is 6–40%,^13^ with similar findings internationally.^12^ This wide variation in prevalence is based upon the definitions of insomnia used, with ‘insomnia symptoms’ showing the highest prevalence and clinically diagnosed chronic insomnia at around 6% prevalence.^12,13^ Prevalence is higher in patients receiving healthcare, with an estimated level of 69% among primary care patients.^14^ Some prior research has sought to identify risk factors associated with poor sleep, from UK Biobank data^5^ and longitudinal or cross-sectional surveys,^15^ and the following were associated with poor sleep: socioeconomic and lifestyle factors (including socioeconomic disadvantage, lower education level, shift work and unhealthy lifestyle), demographic factors (including minority ethnic background, increasing age and female gender) and comorbid illness (including unspecified chronic illness, pain, diabetes, obesity, hypertension, respiratory symptoms, mental illness, substance misuse and menopause).^5,9,15–20^ However, to our knowledge, no prior study has carried out a detailed analysis of medical records to establish the real-world prevalence of insomnia, insomnia-related health inequalities or local unmet need regarding insomnia. This study’s aim was to establish these in North Central London, using routinely collected primary care data.

## Method

### Data extraction and criteria

In the UK, general practice (otherwise known as ‘primary care’) medical records are summarised using codes (previously by the coded system Read^21^ and now by SNOMED^22^), which can be utilised to summarise patient health and clinical interactions, and are useful for extraction of summarised data.

Coded data were extracted from general practice records across three North Central London boroughs (Camden, Islington and Haringey) in December 2021. Data were sourced from a data warehouse, operated by the North East London Commissioning Support Unit to house pseudonymised general practice data. Data were extracted for all patients aged 15+ years and registered with 102 of the 105 local general practices, encompassing 765 035 patients; 3 practices and 9% of patients from these participating practices were excluded due to opting out of data sharing. Data were aggregated at the point of extraction.

From discussions with local general practitioners (GPs), the authors are aware that GPs do not always code for insomnia when a patient presents with insomnia symptoms, perhaps particularly if that patient has also presented with a comorbid illness such as depression, when the depression may be coded instead. Preliminary analysis also supports this issue, because 2553 patients were prescribed tablets from a list of medications used only for insomnia (zopiclone, zolpidem, temazepam and melatonin) without having a code for insomnia. Therefore, the prevalence values sought are estimates.

For the purpose of this study, a patient was considered to have insomnia symptoms if they met one of three criteria:

- criterion 1 – the presence of a code indicating insomnia;
- criterion 2 – the presence of a code indicating insomnia treatment;
- criterion 3 – a sedative medication recently prescribed for sleep.

For criteria 1 and 2, a search was conducted for Read and SNOMED codes containing the following strings: ‘sleep*’, ‘insomn*’, ‘sedat*’ and ‘hypnot*’. L.Z.W. (psychiatrist and insomnia specialist) selected those codes that were relevant to insomnia, and this was cross-checked by another sleep medicine specialist. Codes were excluded that indicated only hypersomnia, sleep apnoea or abnormal behaviours during sleep. A total of 53 codes were identified that indicated an insomnia diagnosis (criterion 1), and 8 codes that indicated treatment for insomnia (criterion 2) (see Appendix 1); 31 codes were excluded (see Appendix 2).

For criterion 3, data were extracted pertaining to hypnotic medications that had been prescribed, and patients were included if those medications were assumed by the authors to have been prescribed for sleep. Based on L.Z.W.’s and the sleep medicine specialist’s clinical experience of which sedative medications are commonly prescribed for sleep in the UK, the following were included:

- temazepam, zopiclone, zolpidem and melatonin (which are usually used only as sleeping tablets);
- promethazine, mirtazapine, amitriptyline and trazadone (which are often given for insomnia, for other disorders or for insomnia comorbid with other disorders).

For patients with the latter prescriptions, they were excluded (i.e. the medication was presumed not to have been prescribed for insomnia) if the patient also had a code recorded on their GP record over the past 5 years for another illness for which that medication was commonly used, as follows (for more details, see Appendix 3):

- promethazine if they also had a code for anxiety or agitation, or were on the Quality and Outcomes Framework (QOF)^23^ register for severe mental illness (SMI);
- mirtazapine or trazadone if they also had a code for anxiety or were on the QOF depression register;
- amitriptyline if they were on the QOF depression register.

Two different estimates were determined: a ‘best estimate’, which determined caseness based upon patients’ histories of being coded as having insomnia, being referred for insomnia treatment or being prescribed medication for insomnia; and a ‘lowest estimate’, which excluded patients whose history of medication for insomnia was less certain. In choosing the criteria, the authors acknowledge that both may be underestimates (see Discussion). Specifically, the following criteria were used:

- ‘Lowest estimate’ prevalence values included patients who had at least one of the following:
  - a code for insomnia over the past 5 years;
  - a prescription of temazepam, zopiclone, zolpidem or melatonin in the past 3 months (these are medications used only as sleeping tablets);
  - a code for CBT-I treatment/referral in the past 6 months;
  - a code for other insomnia treatment, such as sleep hygiene, in the past 5 years.
- ‘Best estimate’ prevalence values additionally included patients who had:
  - a prescription for another medication commonly used for insomnia, without also having a code for a non-insomnia illness for which that medication is a common treatment.

### Rationale for time frames for coded records

Coded records going back 5 years from the date of extraction were included to estimate the current prevalence of insomnia, to strike a balance between inclusion of previous diagnostic activity covering a sufficient period while also having a lower likelihood that the included patients’ insomnia had already resolved. This was also the time frame used for other disorders for which codes were extracted (relating to criterion 3 and to investigation of insomnia in patients with comorbid illness). When considering codes for insomnia treatment as an indication of current prevalence, only codes for CBT-I within the past 6 months were included, since CBT-I is highly effective, and therefore for those with a CBT-I code prior to this, many would have already received treatment and become insomnia free.^4^ However, when considering codes for alternative insomnia treatments (such as sleep hygiene), 5 years was again used as the time frame because research suggests that these treatments are largely ineffective, so patients receiving them can be assumed to still have insomnia.^10^ Sedative medications were considered if these were prescribed within the previous 3 months, because this is the maximum duration of individual prescriptions in UK primary care and, again, for its balance between not being too under- or over-inclusive.

### Extraction of socioeconomic, comorbidity and demographic data

Data were extracted pertaining to body mass index and six other mental and physical health comorbidities, which were selected based on prior knowledge or suspicion of their association with insomnia, and on the feasibility of data extraction. Data relating to area-based deprivation, age, gender and ethnic group were also extracted to allow analysis of variation by these factors. These were all presented as aggregated data for analysis.

### Analysis

Data were analysed using descriptive statistics routinely used by local public health teams in needs analysis. Prevalence was calculated separately for variables pertaining to demographic information and deprivation, which were compared with the mean prevalence for the sample; 95% confidence intervals were used as a test of significance.

## Results

Data were extracted and analysed for 765 035 patients, as described above. Summary tables for the descriptive statistics are shown in Appendix 4.

### Prevalence of insomnia

The ‘best estimate’ for insomnia prevalence was 4.3% (95% CI [4.24, 4.33]) and ‘lowest estimate’ was 3.5% (95% CI [3.45, 3.53]).

We excluded 0.5% of all patients from the list of those prescribed sleeping tablets, despite those individuals having been prescribed medication commonly used for insomnia, due to their having a comorbid condition such as depression, anxiety or SMI.

### Insomnia prevalence by demographic factors and geographical deprivation

**Fig. 1:**
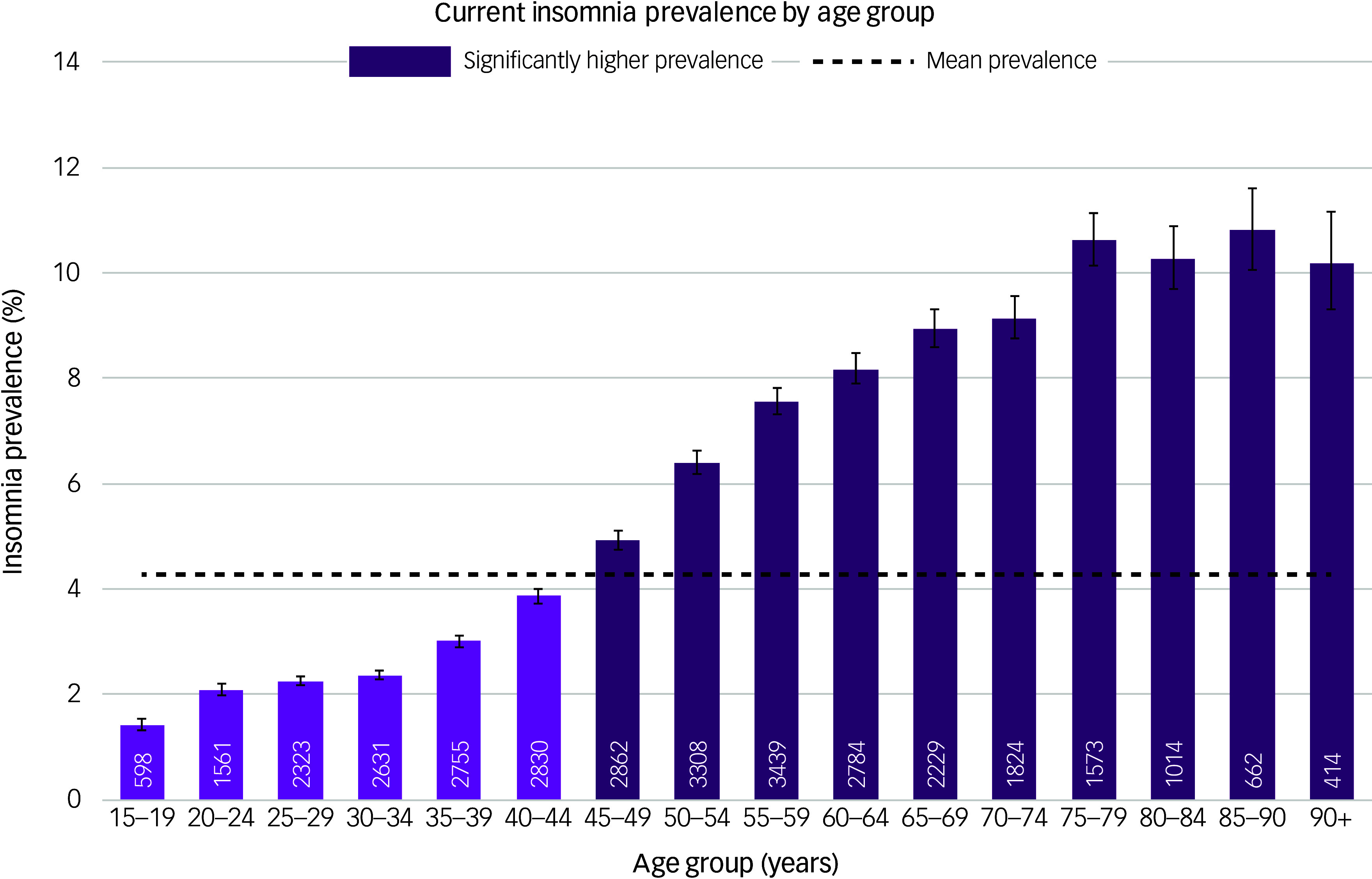
‘Best estimate’ insomnia prevalence by age group.

**Fig. 2:**
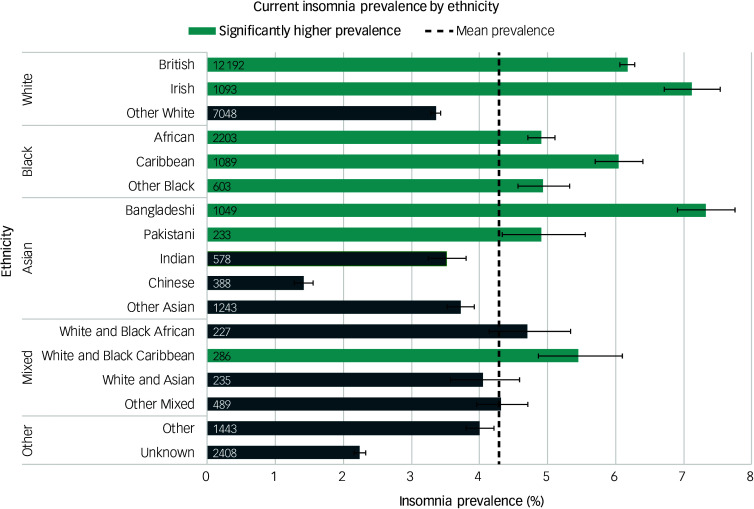
‘Best estimate’ insomnia prevalence by ethnicity.

**Fig. 3:**
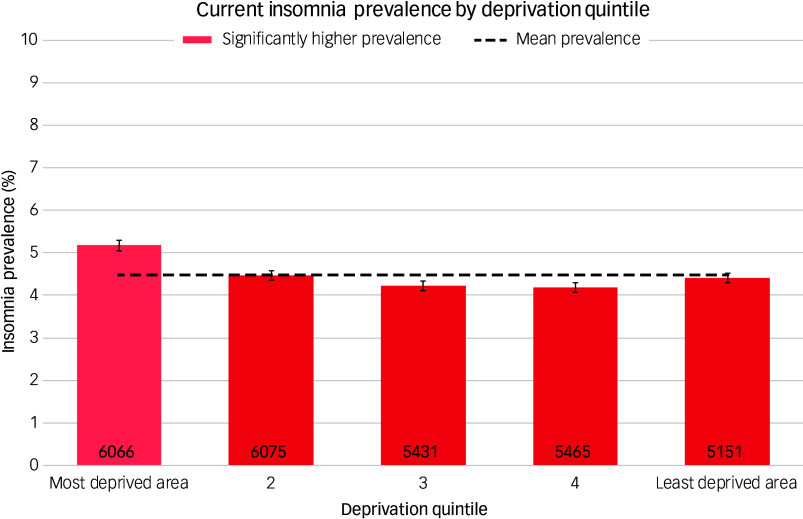
‘Best estimate’ insomnia prevalence by geographical deprivation quintile.

Prevalence was 4.9% for women and 3.7% for men. Prevalence increased steadily with age, being highest in those aged 85–90 years (10.8%, 95% CI [10.1, 11.1]) and lowest in those aged 15–19 years (1.4%, 95% CI [1.3, 1.5]), as shown in Fig. 1. There was significant variation by ethnic group (Fig. 2) and deprivation quintile (Fig. 3), with the highest prevalence being found in the most deprived quintile (5.2%, 95% CI [5.1, 5.3]) and those of Bangladeshi ethnicity (7.3%, 95% CI [6.9, 7.8]).

### Insomnia prevalence by long-term condition

**Fig. 4:**
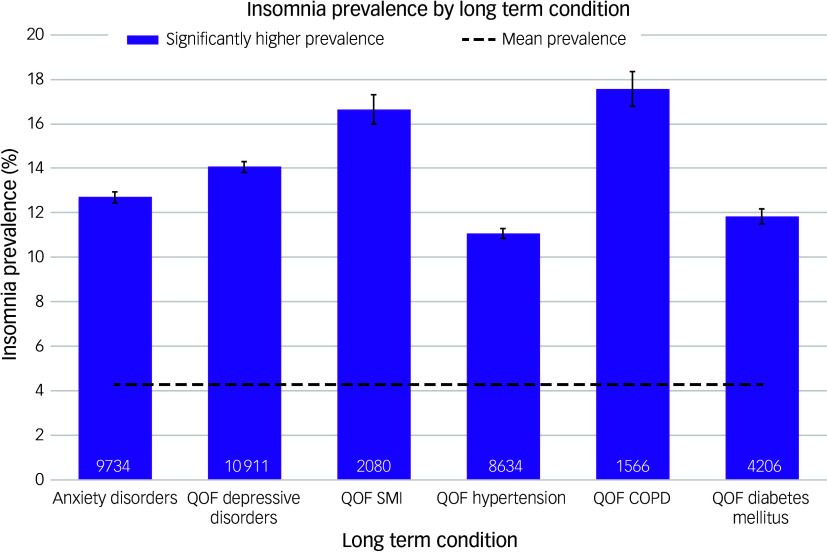
‘Best estimate’ insomnia prevalence by long-term condition. QOF, Quality and Outcomes Framework; SMI, severe mental illness; COPD, chronic obstructive pulmonary disease.

**Fig. 5:**
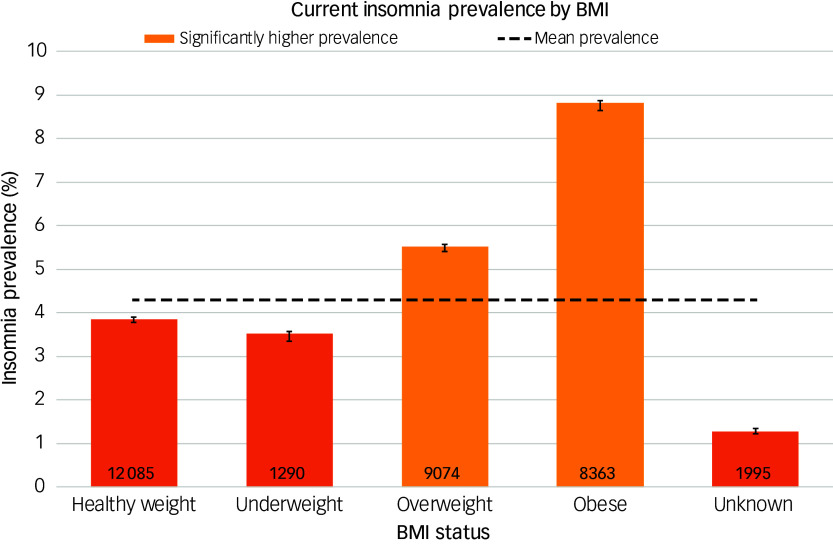
‘Best estimate’ insomnia prevalence by body mass index (BMI).

Insomnia prevalence was significantly higher in patients with any of the six comorbidities for which data were extracted (Fig. 4): chronic obstructive pulmonary disease (COPD; 17.5%, 95% CI [16.8, 18.4]), SMI (16.6%, 95% CI [16.0, 17.3]), depression (14.1%, 95% CI [13.8, 14.3]), anxiety disorders (12.7%, 95% CI [12.5, 12.9]), diabetes mellitus (11.8%, 95% CI [11.5, 12.2]) and hypertension (11.1%, 95% CI [10.8, 11.3]). Prevalence also increased with increasing weight category (Fig. 5), with overweight patients having a higher prevalence (5.5%, 95% CI [5.4, 5.6]) than the mean, and a yet higher prevalence for those in the obese category (8.8%, 95% CI [8.6, 9.0]).

### Treatment rates

It was shown that 1.7% (95% CI [1.2, 2.3]) of people with a code or medication indicating insomnia over the previous 6 months had been referred for CBT-I treatment during that time period, and 5.6% (95% CI [4.7, 6.7]) had a code for another intervention such as sleep hygiene during that time period.

### Variation among general practices

Significant variation in insomnia prevalence occurred between general practices. The highest ‘best estimate’ prevalence was found for the Camden Health Improvement Practice (9.9%, 95% CI [7.5, 12.9]), which is the practice supporting Camden’s homeless population (the other two boroughs do not have a dedicated practice for their homeless populations).

## Discussion

The ‘best estimate’ of insomnia prevalence from North Central London’s primary care data was 4.3%, with the ‘lowest estimate’ not being much lower (3.5%). It is possible that the inclusion of patients based on sedative medications that could also have been used to treat other disorders (such as mirtazapine and promethazine), even when excluding those with certain comorbid conditions, risked the inclusion of some patients who did not have insomnia. Therefore, the ‘lowest estimate’ excluded this criterion within the case definition, to check the extent to which that criterion had impacted the results. The difference between the best and ‘lowest estimate’ was 0.8%, demonstrating that the prevalence was still high even following removal of the criterion of having a sedating medication prescription. As described below, there are multiple reasons why both may actually be underestimates. These findings suggest that insomnia is as common as other illnesses that receive a high share of focus and healthcare resources in many countries.

There was significant variation in prevalence based on the demographic factors investigated. The higher prevalence in women and steady increase with age are consistent with prior research.^9,12,15–19^ Given that insomnia rarely improves spontaneously without treatment once it has become chronic,^5^ and that it is under-treated (both within this sample and nationally^1^), it is understandable that the proportion of people with insomnia would gradually accumulate with increasing age. Increasing rates of insomnia with age may also relate to increasing rates of comorbid illness with age, which warrants further research. Some ethnic groups (especially Bangladeshi, White Irish and Black Caribbean), and those living in the most deprived areas, had a significantly higher prevalence of insomnia compared with the mean. This may reflect health inequalities relating to differences in the prevalence of insomnia, differences in GP access/help-seeking behaviour or the influence of patients’ demographic or socioeconomic factors on how GPs diagnose and code. The Bangladeshi community in this area is known to have generally poor health compared with other groups.^24^ This variation in prevalence by sociodemographic factors (including increasing with sociodemographic disadvantage and age, and being higher in women) is consistent with the broader international literature, suggesting patterns similar to those seen in other countries.^25^

Prevalence was significantly higher in patients with the seven comorbid mental and physical health conditions for which data were extracted (COPD, diabetes mellitus, hypertension, depressive disorders, anxiety disorders, SMI and obesity), consistent with prior research,^5,9,15,16,20^ with the highest prevalence being in those with COPD and SMI. It is likely that other chronic illnesses may also be associated with a high prevalence of insomnia, as well as risk factors such as alcohol misuse. This highlights the importance of actively screening for insomnia in patients with various comorbid disorders. Prior research has suggested that untreated comorbid insomnia renders mental disorders more resistant to treatment and at a higher risk of relapse.^3^ Therefore, particular attention should be paid to screening for, and treating, insomnia in those with mental illness.

Referral rates for CBT-I were low (1.7% for those indicated as having insomnia). It is likely that patients with insomnia who have not been referred for treatment would be less likely to have received an insomnia code, so the proportion of insomnia treated may be even lower. This is concerning, given that CBT-I is the NICE-recommended first-line treatment for insomnia in the UK^7^ and the negative health, quality of life and economic implications of untreated insomnia.^1^ From local knowledge, this is probably due to a combination of limited service availability, unclear referral pathways and competing demands in primary care settings. These findings should be interpreted in the context of the significant pressures facing primary care, where clinicians manage multiple complex needs within limited time. This is also consistent with previous studies conducted in other countries, which highlight the lack of availability and accessibility of CBT-I treatment.^25^

Of those who were referred for therapy, were prescribed sleeping tablets or had other treatments for insomnia (such as sleep hygiene), many did not have a diagnosis code for insomnia, which may reflect variation in clinical coding practices and system-level documentation processes. Furthermore, there was significant variation in the apparent prevalence between practices. This may reflect true variation in local prevalence and/or differences in coding and management approaches between practices.

### Strengths and limitations

A limitation of using GP data to determine disease prevalence is that this relies both on patients presenting to their GP when they have symptoms and on GPs diagnosing and coding problems correctly, and presentation may vary across different patient groups. In this study, all prevalence estimates may be underestimates because not all those with insomnia would have presented to their GP^12,26^ (and many may instead be using self-help or over-the-counter treatments);^26^ in addition, it is likely that insomnia is under-coded and under-treated in routine primary care, reflecting both service limitations and the many competing demands faced by clinicians. Additionally, prevalence estimates excluded those without an insomnia code who were prescribed sleeping tablets but had these stopped within the past 3 months; those who have died or moved out of the area since presenting with insomnia; and those taking less commonly prescribed sleeping tablets (e.g. diazepam). Furthermore, they excluded people for whom sedative medication was prescribed that could have been intended for a different mental illness (0.8%), even though insomnia commonly exists alongside other mental illnesses (meaning that many of these excluded patients may have actually been prescribed those medications for insomnia). Another limitation of this study is that free text could not be accessed to confirm prevalence, and patients were counted as having insomnia only if this could be identified from coded data or prescription information. This restricted insight into the severity or duration of patients’ insomnia symptoms, or whether they met criteria for a chronic insomnia diagnosis, and therefore whether CBT-I would have been indicated. The availability of solely aggregated data limited the complexity of the analysis that could be carried out, including the possibility of any regression analysis.

However, this study also has a number of strengths. It analysed a very substantial data-set across three large boroughs – the largest study the authors have identified that investigated insomnia prevalence or insomnia-related health inequality. With a large sample size also comes higher generalisability to the rest of the UK and international populations. To partially overcome the challenges associated with determining insomnia prevalence from the data, two different prevalence estimates were provided (‘best estimate’ and ‘lowest estimate’), which demonstrated that even when most sedative medications were removed from case-finding, the prevalence was found to be high.

### Recommendations

The high prevalence and low treatment rates found in this study demonstrate the importance of increasing treatment rates. Under-treatment of insomnia is likely to be a national problem,^1^ and has also been reported in other countries;^8–10,25^ therefore, the recommendations given below are likely to be relevant outside of North Central London.^8–10^ Given the health economic impact of untreated insomnia and the cost-effectiveness of CBT-I, allocating resources to increasing effective treatment is warranted to save money elsewhere.^2^

Increasing treatment rates requires a combination of a greater number of referrals for appropriate treatment and greater availability of accessible treatment services. While CBT-I is currently available to all patients in the UK via remote sessions from a small number of sleep clinics that can be accessed regardless of address,^27,28^ service capacity will need to be expanded to accommodate referral numbers as they increase. Currently, some National Health Service (NHS) Talking Therapy (formally called ‘IAPT’) services provide CBT-I, but this is a postcode lottery since insomnia is not included in the NHS Talking Therapies Manual of national service requirements.^29^

As such, the following measures are recommended.

### Training

- Mental health and primary care clinicians may benefit from attending training courses or webinars on insomnia management (which may not always have been included in medical and specialty training curricula), and this topic should be added to curricula in the future. Brief strategies from CBT-I can then be relayed to patients during their consultations, while they are awaiting formal CBT-I.
- To expand the availability of trained CBT-I therapists, healthcare professionals working in primary care and mental health services should be encouraged to undergo more comprehensive training to deliver formal CBT-I, although this training is still not time-intensive: for example, some UK sleep clinics run 1- to 3-day courses.^1^ Non-clinicians, such as psychological well-being practitioners (PWPs) working in NHS Talking Therapy services, could also be trained to deliver CBT-I under the supervision of qualified therapists, as has been done successfully previously.^30^
- Facilitating clinician familiarity with CBT-I services and local referral pathways may help strengthen treatment delivery in routine care.^1^

### Detection of chronic insomnia

- Clinicians in primary and secondary care should actively enquire about insomnia, especially in regard to those with advancing age, socioeconomic deprivation, mental illness, obesity, diabetes mellitus and respiratory disease – groups for which the prevalence was shown to be up to 20% in this study. This active enquiry is important, since research suggests that up to 40–45% of individuals with insomnia do not make their clinicians aware of this.^12,26^
- Responses to initial screening questions, such as ‘Do you have problems with falling or staying asleep?’ and those about frequency, chronicity, daytime impairment and sleeping medication use, could then prompt the use of standardised questionnaires, such as the Insomnia Severity Index,^31^ and screening for comorbid sleep disorders.^32^

### Treatment pathways

- Clear local insomnia treatment pathways should be established, differentiating between clinicians in primary and secondary care. Following this study, a pathway was created in North Central London by an Insomnia Working Group (comprising GPs, sleep specialists and pharmacists), for which the group has given permission to be adapted and used elsewhere.^32^

### Chronic insomnia treatment services

- CBT-I treatment services should be commissioned so that they are accessible to patients nationally. The probable inequalities in insomnia related to factors such as deprivation and ethnicity should be acknowledged, and attention should be paid to designing and running services that facilitate equitable access, including consideration of the location and cultural appropriateness of services.
- As with other common mental disorders, a stepped-care model is recommended,^1,8^ such as:
  - Step 1: self-help or guided self-help – for example, the Sleepio app is NICE-recommended and cost-effective^33,34^ but has high drop-out rates and is not suitable for everyone.^35^ At the time of writing, the Sleepful app,^36^ developed by the Clinical Sleep Research Unit at Loughborough University (UK), is freely available and recommended by some sleep experts, although it has not undergone clinical trials.
  - Step 2: therapist-led individual or group CBT-I (e.g. in talking therapies services and secondary care mental health services).
  - Step 3: referral to a specialist sleep clinic.
    In a stepped-care model, patients would start off at step 1 and move up the steps if previous steps are ineffective. Only those with a clear need for specialist input would enter the pathway at a higher step, making the model more cost-effective.
- To enable this, chronic insomnia should be incorporated into the NHS Talking Therapies Manual of national service requirements,^29^ so that CBT-I becomes a core treatment offered by all Talking Therapies services. This would help eliminate the current national postcode lottery and ensure that specialist sleep clinics are reserved for patients with more complex needs.^1^
- Medication for insomnia may be considered at different steps in treatment – either combined with or as a second-line alternative to CBT-I – as described in the North Central London Insomnia in Adults Clinical Pathway.^32^

### Population health management and coding

- Screening and management of insomnia could be supported by embedding it within population health management registries – for example, those focused on ageing well, mental illness and multimorbidity. Additional prompts, such as markers or pop-ups within GP clinical systems, may also help prompt identification and intervention.
- Efforts to support consistent coding approaches among practices may improve patient care and enable better system-wide population health management and research capabilities. The list of current insomnia-related SNOMED codes shown in Appendix 1 may provide a useful starting point; however, the range of insomnia-related codes available to GPs is extensive, and none clearly distinguish between acute and chronic insomnia – a distinction that is essential, because the treatment for chronic insomnia (a recognised mental disorder) differs from that for brief, time-limited insomnia.^5,32^ Therefore, coding and system prompts should be refined to support this differentiation.

### Public health strategies

- There is increasing evidence that, for those with chronic insomnia, CBT-I has a role in primary, secondary and tertiary prevention of comorbid disorders,^1^ such as depression^37–39^ and asthma.^40^ Therefore, public health bodies should consider insomnia as part of Joint Strategic Needs Assessments. Insomnia should be included within national and local prevention strategies, in keeping with the UK NHS Long Term Plan.^41^

### Future research

- Future research that includes access to patient-level data at the analysis stage may be beneficial, especially if it were to make use of artificial intelligence, such as natural language processing, to identify and confirm cases from the free text available. Such analysis would enable more detailed exploration into how and why prevalence varies by demographic factors, deprivation and comorbidity, the timeline of when insomnia is reported in relation to comorbidity and the association between insomnia and other health conditions. Further research looking at associations between demographic or deprivation factors and diagnosis and treatment rates could establish how, and the extent to which, health inequalities have contributed towards variation between general practices.
- The current study could be repeated following implementation of some of the above recommendations, and replicated in other regions of the UK and internationally.

## Data Availability

The non-identifiable data were accessed as part of an National Health Service (NHS) data-sharing agreement for public health analysis, and the authors no longer have access to the disaggregated raw data that were used. However, the aggregated data used were a result of data processing carried out by U.O. and these data will be kept for a minimum of 5 years.

## Appendix 1: List of SNOMED Clinical Terms (CTs) included (and their associated Read Code Version 2)

**Insomnia codes:**

**Table:**

Read Code Version 2 (V2)	v2 Term Code	v2 Term30	SNOMED CT ConceptID	SNOMED CT DescriptionID	SNOMED CT Term
1B1B.	00	Cannot sleep - insomnia	193462001	297924011	Insomnia
1B1B.	11	C/O - insomnia	272025006	2669520013	Complaining of insomnia
1B1B0	00	Initial insomnia	59050008	98133013	Initial insomnia
1B1B1	00	Middle insomnia	67233009	111701012	Middle insomnia
1B1B2	00	Late insomnia	162204000	252935019	Late insomnia
E274.	12	Insomnia-nonorg.sleep disorder	192454004	296381018	Nonorganic insomnia
E2741	00	Transient insomnia	268652009	401805019	Transient insomnia
E2741	11	Insomnia NOS	193462001	297924011	Insomnia
E2742	00	Persistent insomnia	191997003	295412011	Persistent insomnia
Eu510	00	[X]Nonorganic insomnia	192454004	296381018	Nonorganic insomnia
R005.	11	[D]Insomnia - symptom	193462001	297924011	Insomnia
R0051	00	[D]Insomnia with sleep apnoea	41975002	197601019	Insomnia with sleep apnoea
R0052	00	[D]Insomnia NOS	193462001	297924011	Insomnia
1B1Q.	00	Poor sleep pattern	314938000	459355013	Poor sleep pattern
1BX0.	00	Delayed onset of sleep	401161007	1780361011	Delayed onset of sleep
1BX9.	00	Light sleep	29373008	49145012	Light sleep
E274.	00	Non-organic sleep disorders	270487001	405106013	Non-organic sleep disorder
E274.	12	Insomnia-nonorg.sleep disorder	192454004	296381018	Nonorganic insomnia
E2740	00	Non-organic sleep disord.unsp.	270487001	405106013	Non-organic sleep disorder
E2746	00	Shifting sleep-work schedule	713498009	3289614015	Circadian rhythm sleep disorder of shift work type
E274B	00	REM-sleep type	192004002	295422017	Repeated rapid eye movement sleep interruptions
E274C	00	Other sleep stage/arousal dysf	39898005	67285010	Sleep disorder
E274D	00	Repetitive intrusions of sleep	268654005	401807010	Repetitive intrusions of sleep
E274D	11	Restless sleep	12262002	21110011	Restless sleep
E274F	00	Inversion of sleep rhythm	192008004	295430016	Inversion of sleep rhythm
E274y	00	Other non-org.sleep disorder	270487001	405106013	Non-organic sleep disorder
E274z	00	Non-organic sleep disorder NOS	270487001	405106013	Non-organic sleep disorder
Eu51.	00	[X]Nonorganic sleep disorders	270487001	405106013	Non-organic sleep disorder
Eu512	00	[X]Nonorgan dis of sleep-wake	268722008	401890019	Non-organic disorder of the sleep-wake schedule
Eu512	11	[X]Psychogen inv/circad rhythm	268722008	401890019	Non-organic disorder of the sleep-wake schedule
Eu512	12	[X]Psych invers/nyctohem rhyth	268722008	401890019	Non-organic disorder of the sleep-wake schedule
Eu512	13	[X]Psychogen inver/sleep rhyth	268722008	401890019	Non-organic disorder of the sleep-wake schedule
Eu51y	00	[X]Other nonorganic sleep dis	270487001	405106013	Non-organic sleep disorder
Eu51z	00	[X]Nonorganic sleep dis unsp	270487001	405106013	Non-organic sleep disorder
Eu51z	11	[X]Emotional sleep disord NOS	270487001	405106013	Non-organic sleep disorder
Fy0..	00	Sleep disorders	39898005	67285010	Sleep disorder
Fy00.	00	Disordr/initiat&maintain sleep	194437008	299274011	Disorders of initiating and maintaining sleep
Fy02.	00	Disorders/sleep-wake schedule	271794005	406739012	Disorders of the sleep-wake schedule
Fyu58	00	[X]Other sleep disorders	39898005	67285010	Sleep disorder
R005.	00	[D]Sleep disturbances	44186003	493802012	Sleep disturbance
R005.	12	[D]Sleep rhythm problems	3745000	1229316019	Sleep rhythm problem
R0050	00	[D]Sleep disturbance, unspecif	44186003	493802012	Sleep disturbance
R0051	00	[D]Insomnia with sleep apnoea	41975002	197601019	Insomnia with sleep apnoea
R0055	00	[D]Sleep rhythm inversion	192008004	295428018	Reversed sleep-wake cycle
R0056	00	[D]Sleep rhythm irregular	271793004	406735018	Irregular sleep-wake pattern
R0057	00	[D]Sleep-wake rhythm non-24-hr	230496009	345393016	Non-24 hour sleep-wake cycle
R0058	00	[D]Sleep dysfunct./sleep stage	441877007	2818183012	Sleep dysfunction with sleep stage disturbance
R0059	00	[D]Sleep dysfunction/arousal	442176004	2819608011	Sleep dysfunction with arousal disturbance
R005z	00	[D]Sleep dysfunction NOS	39898005	67285010	Sleep disorder
ZV1B1	00	[V]Per hst/unhlth slp-wake scd	271794005	406736017	Disorder of sleep-wake cycle
1BX3.	00	Early morning waking	401236004	1780430015	Early morning waking
E274E	00	Short-sleeper	192007009	295427011	Short-sleeper

**Insomnia treatment codes:**

**Table:**

Read Code Version 2 (V2)	V2 TermCode	V2 Term30	SNOMED CT ConceptID	SNOMED CT DescriptionID	SNOMED CT Term
8G98.	00	Stimulus control behavior educ	440313002	2795162013	Education about stimulus control behaviour in insomnia
8G99.	00	Sleep restriction therapy	440089000	2793349016	Sleep restriction therapy
8G9B.	00	Sleep hygiene behavior educatn	439035007	2792682019	Education about sleep hygiene behaviour
8Q0..	00	Sleep management	395069004	1488768014	Sleep management
–	–	–	1107191000000102	2770731000000119	Referral for digital cognitive behavioural therapy for insomnia
–	–	–	1107201000000100	2770771000000117	Self referral for digital cognitive behavioural therapy for insomnia
–	–	–	868185009	3963816014	Cognitive behavioural therapy for insomnia
–	–	–	1109721000000108	2776901000000113	Digital cognitive behavioural therapy for insomnia

## Appendix 2: List of SNOMED Clinical Terms (CTs) excluded (and their associated Read Code Version 2)

**Table:**

Read Code Version 2 (V2)	v2 TermCode	v2 Term30	SNOMED CT ConceptID	SNOMED CT DescriptionID	SNOMED CT Term
1B1R.	00	Good sleep pattern	314939008	459356014	Good sleep pattern
1BX..	00	Sleep observations	118190003	1220362019	Sleep observations
1BX1.	00	Excessive sleep	77692006	1234153012	Excessive sleep
38D0.	00	Pittsburgh sleep quality index	699200007	2983135015	Pittsburgh sleep quality index
38Da.	00	Berlin questionnr sleep apnoea	445483007	2871082010	Berlin questionnaire for sleep apnoea
663N.	00	Asthma disturbing sleep	170631002	264540018	Asthma disturbing sleep
663N1	00	Asthma disturbs sleep weekly	170633004	264542014	Asthma disturbs sleep weekly
663N2	00	Asthma disturbs sleep freqntly	170634005	264543016	Asthma disturbs sleep frequently
663O.	00	Asthma not disturbing sleep	170635006	264544010	Asthma not disturbing sleep
663O0	00	Asthma never disturbs sleep	170636007	264545011	Asthma never disturbs sleep
66Yg.	00	COPD disturbs sleep	198401000000104	299001000000116	Chronic obstructive pulmonary disease disturbs sleep
66Yh.	00	COPD does not disturb sleep	198411000000102	299031000000110	Chronic obstructive pulmonary disease does not disturb sleep
67IT.	00	Advi to carer re childs sleep	408434007	2159996010	Advice to carer regarding child’s sleep
E2747	00	Somnambulism - sleep walking	80495009	2951689017	Sleepwalking
E274A	00	Sleep drunkenness	192003008	295421012	Sleep drunkenness
Eu514	00	[X]Sleep terrors	89675003	508626017	Sleep terrors
F13z5	00	Benign neonat sleep myoclonus	413638006	2532986018	Benign neonatal sleep myoclonus
F28yz	11	Ondine’s curse	230499002	345396012	Sleep-related respiratory failure
Fy03.	00	Sleep apnoea	73430006	200354014	Sleep apnoea
Fy03.	11	Obstructive sleep apnoea	78275009	2966563014	Obstructive sleep apnoea
Fy04.	00	Sleep-related respirat failure	230499002	345396012	Sleep-related respiratory failure
Fy04.	11	Ondine’s curse	230499002	345396012	Sleep-related respiratory failure
Fy05.	00	Noct sleep-related eating dis	407666007	2159265012	Nocturnal sleep-related eating disorder
H5B..	00	Sleep apnoea	73430006	200354014	Sleep apnoea
H5B0.	00	Obstructive sleep apnoea	78275009	2966563014	Obstructive sleep apnoea
Q318.	00	Prim sleep apnoea of newborn	361208003	477724014	Primary sleep apnoea of newborn
R0053	00	[D]Hypersomnia + sleep apnoea	79280005	200785019	Hypersomnia with sleep apnoea
R0053	11	[D]Sleep apnoea syndrome	502901000000102	1112961000000111	[D]Sleep apnoea syndrome
R0053	12	[D]Syndrome sleep apnoea	502901000000102	1112961000000111	[D]Sleep apnoea syndrome
U607z	12	[X] Adv react sleep pill NOS	292331008	432467017	Sedative adverse reaction

## Appendix 3: SNOMED codes to identify comorbid mental illness

- Anxiety: a list of codes for anxiety were taken from https://clinicalcodes.rss.mhs.man.ac.uk/medcodes/article/78/.^42^
- Agitation: SNOMED Clinical Term ‘Restlessness and agitation’ (Concept Code ID: 274647009).
- Codes indicating severe mental illness, depression, chronic obstructive pulmonary disease, hypertension and diabetes mellitus were taken from the list of codes from the relevant Quality Outcomes Framework (QOF) disease registry business rules.^23^

## Appendix 4: Summary tables for descriptive statistics

**Table 4a:** ‘Best estimate’ insomnia prevalence by age group

Age group (years)	‘Best estimate’ for the number of insomnia cases	Total in age group	Percentage
15–19	598	42 212	1.42
20–24	1561	74 697	2.09
25–29	2323	103 155	2.25
30–34	2631	111 198	2.37
35–39	2755	91 503	3.01
40–44	2830	73 147	3.87
45–49	2862	58 154	4.92
50–54	3308	51 692	6.40
55–59	3439	45 468	7.56
60–64	2784	34 069	8.17
65–69	2229	24 920	8.94
70–74	1824	19 950	9.14
75–79	1573	14 808	10.62
80–84	1014	9877	10.27
85–90	662	6123	10.81
90+	414	4062	10.19

**Table 4b:** ‘Best estimate’ insomnia prevalence by ethnicity

Broad ethnic group	Ethnicity	‘Best estimate’ for the number of insomnia cases	Total in ethnic group	Percentage
White	British	12 192	197 542	6.17
	Irish	1093	15 360	7.12
	Other White	7048	210 014	3.36
Black	African	2203	44 929	4.90
	Caribbean	1089	18 026	6.04
	Other Black	603	12 232	4.93
Asian	Bangladeshi	1049	14 329	7.32
	Pakistani	233	4747	4.91
	Indian	578	16 442	3.52
	Chinese	388	27 438	1.41
	Other Asian	1243	33 428	3.72
Mixed	White and Black African	227	4826	4.70
	White and Black Caribbean	286	5249	5.45
	White and Asian	235	5797	4.05
	Other mixed	489	11 330	4.32
Other	Other	1443	36 025	4.01
	Unknown	2408	107 321	2.24

**Table 4c:** ‘Best estimate’ insomnia prevalence by geographical deprivation quintile

Deprivation quintile	‘Best estimate’ for the number of insomnia cases	Total in area	Percentage
Most deprived area (1)	6066	117 314	5.17
2	6075	135 938	4.47
3	5431	128 463	4.23
4	5465	130 851	4.18
Least deprived area (5)	5151	116 844	4.41
Out of borough	4558	134,246	3.40
Area of residence unknown	61	1379	4.42
Total	28 188	629 410	4.48

**Table 4d:** ‘Best estimate’ insomnia prevalence by long-term condition (LTC)

LTC	‘Best estimate’ for the number of insomnia cases	Total in LTC group	Percentage
Anxiety disorders	9734	76 668	12.70
QOF depressive disorders	10 911	77 590	14.06
QOF SMI	2080	12 514	16.62
QOF hypertension	8634	78 095	11.06
QOF COPD	1566	8926	17.54
QOF diabetes mellitus	4206	35 553	11.83

QOF, Quality and Outcomes Framework; SMI, severe mental illness; COPD, chronic obstructive pulmonary disease.

**Table 4e:** ‘Best estimate’ insomnia prevalence by body mass index (BMI)

BMI group	‘Best estimate’ for the number of insomnia cases	Total in BMI group	Percentage
Underweight	1290	36 640	3.52
Healthy weight	12 085	313 456	3.86
Overweight	9074	164 469	5.52
Obese	8363	94 848	8.82
Unknown	1995	155 622	1.28

**Table 4f:** ‘Best estimate’ insomnia prevalence by gender

Gender	‘Best estimate’ for the number of insomnia cases	Total in group	Percentage
Female	18 784	383 338	4.90
Male	14 023	381 661	3.67

**Table 4g:** ‘Best estimate’ insomnia prevalence in all patients

	‘Best estimate’ for the number of insomnia cases	Total number of people registered at time of extraction (7 Feb 2022)	Percentage
All patients	32 807	765 035	4.3
